# Prevalence and predictors of malignancies in a polycentric cohort of HIV patients from Italy

**DOI:** 10.7448/IAS.17.4.19652

**Published:** 2014-11-02

**Authors:** Elena Mazzotta, Monica Tontodonati, Chiara Gabrielli, Susanna Mazzocato, Marcello Mazzetti, Katia Falasca, Giovanni Cenderello, Jacopo Vecchiet, Francesco Barchiesi, Daniela Francisci, Giustino Parruti

**Affiliations:** 1 Infectious Diseases Unit, Pescara General Hospital, Pescara, Italy; 2 Infectious Diseases Clinic, Università degli Studi di Perugia, Perugia, Italy; 3 Infectious Diseases Clinic, Department of Biomedical Science, Università Politecnica delle Marche, Ancona, Italy; 4 Infectious Diseases Unit, AOU Carreggi, Firenze, Italy; 5 Infectious Diseases Clinic, Università “G. D'Annunzio”, Chieti, Italy; 6 Infectious Diseases Unit, Galliera Hospital, Genova, Italy

## Abstract

**Introduction:**

HIV infected patients have a higher risk of developing cancer than the general population. Kaposi's sarcoma, non-Hodgkin's lymphoma, primary CNS lymphoma and invasive cervical cancers are considered as AIDS defining [1]. An increased incidence in recent years has been reported also for other malignancies after the introduction of cART [2,3].

**Materials and Methods:**

We performed a retrospective multicentric evaluation of all HIV infected patients with both AIDS and non-AIDS defining neoplasms at six Infectious Disease Units spread throughout Italy since 1991 through 2013. Cases were compared with equal number of controls without neoplasia followed at the same institutions, matched for length of HIV infection.

**Results:**

Since 1991, 339 consecutive cases of malignancy were collected from the six convening centres, including approximately an equal proportion of AIDS (51.2%) and non-AIDS defining tumours. Mean prevalence of tumours among centres was 8.3% (r. 6.1%–9.6%). Mean age at tumour diagnosis was significantly lower than in controls (42.6±11.0 vs 46.8±10.6 years, respectively, p<0.0001). As to risk factors for HIV infection, approximately 1/4 (26.1%) of patients were drug abusers, in equal proportion as in controls. A remarkable higher proportion of cancer patients had CD4 T-cell counts <200 c/mmc at time of diagnosis (45.2% vs 13.3%, p<0.0001). Seventy percent of tumours occurred in males; 52.8% of tumour patients were diagnosed with AIDS before and 19.0% at the time of tumour diagnosis. Ninety (28.1%) tumour patients were dead at the time of data collection, a much higher proportion than among cases (12.9%, p<0.0001). Deaths among non-AIDS (20.8%) and AIDS defining tumour patients (35.0%) were significantly different (p=0.005). Predictors of AIDS defining tumours at the time of data collection were: male sex (57.9% vs 40.6%, p=0.004), CD4 T-cell counts <200 c/mmc (63.6% vs 44.1%, p<0.0001), whereas being cART treated at the time of tumour diagnosis was protective (38.0% vs 68.0%, p<0.0001). In the final multivariate model of logistic regression, male sex (OR=2.0, p=0.03) and not being cART treated (OR=2.5, p=0.001) held as independent predictors.

**Conclusions:**

Our retrospection revealed a considerably high proportion of non-AIDS defining tumours, apparently at rise in recent years. We registered high prevalence of tumours in each centre. Absence of cART seemed related with AIDS defining tumours: once more prevention of late presentation appeared the way to avoid worse prognosis in this setting.
